# Hypovolemic Status in Older Hip Fracture Patients Elucidated by Preoperative Transthoracic Echocardiography

**DOI:** 10.1155/2021/9243945

**Published:** 2021-10-04

**Authors:** Yasuhiro Watanabe, Toru Kaneda

**Affiliations:** Department of Anesthesia, Japanese Red Cross Shizuoka Hospital, Shizuoka 420-0853, Japan

## Abstract

Older patients undergoing hip fracture surgery often experience intraoperative hemodynamic instability despite maintaining cardiac function. Although preoperative hemodynamics in such patients have been demonstrated mainly through invasive monitoring, few studies have addressed hemodynamics based on noninvasively measured parameters. We aimed to investigate preoperative hemodynamic states in older hip fracture patients using transthoracic echocardiography (TTE). The TTE data of patients aged >75 years who underwent hip fracture surgery or elective total hip arthroplasty (THA) between April 1, 2019, and March 31, 2021, were collected. In addition to the baseline characteristics, the TTE data from hip fracture patients were compared with the data of those who underwent THA. The hip fracture patients (*n* = 167) were significantly older and had lower stroke volume (45.6 vs. 50.9 ml; *p* < 0.01) and stroke index (33.7 vs. 36.6 ml/m^2^; *p* < 0.01) compared to those who underwent elective THA (*n* = 44). However, the cardiac output (3.51 vs. 3.48 L/min; *p*=0.273) and cardiac index (2.6 vs. 2.47 L/min/m^2^; *p*=0.855) for both groups were almost identical due to the increase in heart rate in the hip fracture group. Regarding other parameters including ejection fraction, fractional shortening, E/E′, and inferior vena cava diameter, there were no significant differences between the two groups. Our noninvasive TTE investigations suggested that hip fracture patients were volume-depleted, and the hypovolemic status activated the sympathetic nervous system, compensating for their cardiac output. Anesthesiologists must focus on the TTE-assessed parameters reflecting the volume status along with the cardiac function.

## 1. Introduction

Hip fractures are a worldwide public health concern, with high morbidity and mortality among older individuals [1]. Although a large study demonstrated that preoperative transthoracic echocardiography (TTE) was not associated with a reduction of in-hospital mortality or postoperative complications in hip fracture patients [2], recent guidelines still acknowledge that TTE can help quantify the nature of valvular heart disease and the degree of cardiac impairment [3]. Thus, TTE's applicability should be assessed for individual patients, depending on patient conditions. Older patients undergoing hip fracture surgery often experience intraoperative hemodynamic instability in spite of maintaining cardiac function. Although preoperative hemodynamics in such patients have been demonstrated through invasive monitoring [4, 5] and noninvasive technology [6], few studies have discussed the preoperative hemodynamic status based on TTE-assessed parameters. Hence, we aimed to investigate preoperative hemodynamics in older patients undergoing hip surgery using TTE.

## 2. Materials and Methods

This retrospective study was approved by the Ethics Committee of the Japanese Red Cross Shizuoka Hospital (approval number: 2021-01), and the requirement for informed consent was waived.

### 2.1. Data Collection

The TTE data of patients aged >75 years who underwent hip (femoral neck and trochanteric) fracture surgery or elective total hip arthroplasty (THA) between April 1, 2019, and March 31, 2021, were collected. The TTE data from hip fracture patients who received TTE examination within 48 h of the injury were compared with the data of those who underwent THA. In addition to patients' baseline characteristics, intake of beta-blockers, anticoagulants, and antiplatelet drugs was recorded. Furthermore, comorbidities which presumably influence the TTE-assessed parameters were investigated.

TTE-assessed parameters reflecting volume status such as left ventricular end diastolic volume (EDV) and end systolic volume (ESV) were calculated with the 2D Teichholz method [7], as were the ejection fraction (EF) and fractional shortening (FS). Stroke volume (SV) was determined by subtracting ESV from EDV, and cardiac output (CO) was calculated based on the heart rate (HR) during the examination (CO = SV × HR). The SV and CO were divided by the body surface area to obtain the stroke index (SI) and cardiac index (CI), respectively. E/E′ which is defined as the ratio of early mitral valve flow velocity (E) to early diastolic mitral annular velocity (E′) and inferior vena cava (IVC) diameter were also recorded.

### 2.2. Statistical Analysis

Continuous variables were presented as median (interquartile range) and compared using the Mann–Whitney *U* test or unpaired *t*-test as indicated. Categorical variables were presented as numbers (percentage) and compared using Fisher's exact test. All statistical analyses were performed with the Prism software version 7 (GraphPad Software, La Jolla, CA, US), and a *p* value <0.05 was considered statistically significant.

## 3. Results and Discussion

Table 1 provides the demographic characteristics of the two groups. Hip fracture patients were significantly older than those undergoing THA (87 vs. 82.5 years; *p* < 0.01) and had significantly lower bodyweight and body surface area. The incidence of each medication and comorbidity was not significantly different between the two groups. Both hemoglobin and hematocrit levels prior to the TTE examination were significantly lower in the hip fracture group than in the THA group.

Table 2 provides the echocardiographic findings of the two groups. The SV (45.6 vs. 50.9 ml; *p* < 0.01) and SI (33.7 vs. 36.6 ml/m^2^; *p* < 0.01) values for the hip fracture group were significantly (as much as 10%) lower than those for the THA group. However, the CO (3.51 vs. 3.48 L/min; *p*=0.273) and CI (2.6 vs. 2.47 L/min/m^2^; *p*=0.855) values for both groups were almost identical, presumably due to the compensatory increase in the HR of the hip fracture patients (78 vs. 66.5 beats/min; *p* < 0.01). Both EF and FS values in the hip fracture group were equal to those in the THA group. E/E′ and IVC diameter value, which correlated with left ventricular diastolic function [8] and venous return, respectively, did not significantly differ between the two groups.

To our knowledge, this is the first report which delineated the preoperative hemodynamic status in hip fracture patients by using TTE-assessed parameters. Patients undergoing elective THA were selected as controls, since it was considered appropriate to compare the data of patients who had surgery on similar sites. However, because of its retrospective nature, the patient characteristics such as sample size, age, and body size differed between the groups. To compare the two groups, we converted SV and CO into SI and CI, respectively. The SI (median: 33.7 mL/m^2^) and CI (median: 2.6 L/min/m^2^) for the hip fracture patients in the present study (Table 2) were comparable to previously reported values determined through invasive monitoring [4], wherein the mean SI was approximately 30 mL/m^2^ and the CI was 2.6 L/min/m^2^, partially supporting our data' validity. We demonstrated that SV and SI in the hip fracture group were significantly lower than those in the THA group. Given the significantly lower left ventricular EDV and ESV along with the comparable systolic function in the hip fracture patients (Table 2), it seems reasonable to consider that they had a smaller amount of circulating blood volume compared to the THA patients. On the other hand, there was no significant difference in IVC diameter between the two groups. The IVC diameter reflects venous return and right atrial pressure [9], and the changes can be good indicators of fluid status, especially in a chronological comparison of the same case. However, the IVC diameter is affected by multiple comorbidities including congestive heart failure, valvular diseases, arrhythmias, and lung diseases [10]; hence, a comprehensive interpretation is essential along with other parameters and pathological conditions. In the present study, the higher incidence of right-sided valvular diseases in the hip fracture group, although not being significant, may have contributed to the result.

Fracture pain may influence sympathetic nerve activity; however, elderly patients with hip fracture often complain of no pain unless the fractured limb is moved by an external force and are treated with analgesics if they cannot tolerate the pain. Thus, the hypovolemic status because of bleeding and inadequate fluid administration was primarily thought to be attributable to the compensatory increase in the HR of the hip fracture group. SV and SI were approximately 10% lower in the hip fracture group than those in the THA group. These findings indicate that a significant amount of crystalloid is required to restore the loss of circulating blood volume along with the maintenance fluid administration in hip fracture patients. Both general and regional anesthesia invariably suppress sympathetic nerve activity and may unmask hypovolemia in such patients.

Our study had certain limitations. First, patients receiving beta-blockers, anticoagulants, and antiplatelet drugs and those with comorbidities were included in our study (Table 1). These conditions may affect the TTE-assessed parameters; however, the incidence of either condition was not significantly different between both groups. Thus, we think that our analyses should reflect real-world settings. Second, we included the data of hip fracture patients who received TTE examination within 48 h of the injury. It was practically difficult for all hip fracture patients to be examined within 24 h of the injury; on the other hand, it was considered that TTE-assessed parameters would be modified in a longer period of time after the injury.

## 4. Conclusion

Our noninvasive TTE investigations suggest that older hip fracture patients were volume-depleted and tachycardic with compensated CO and CI. Anesthesiologists must focus on the TTE-assessed parameters reflecting the volume status along with the cardiac function.

## Figures and Tables

**Table 1:** Demographic characteristics of patients with hip fracture and those who underwent total hip arthroplasty (THA).

	Hip fracture (*n* = 167)	THA (*n* = 44)	*P*
Age (year)	87.0 (83 − 91)	82.5 (78 − 86)	<0.01
Female	139 (83.2)	35 (79.5)	0.656
Height (cm)	150 (145 − 154)	147 (140 − 153)	0.180
Body weight (kg)	44.0 (39.2–51.0)	52.1 (43.6–59.6)	<0.01
Body surface area (m^2^)	1.34 (1.26–1.46)	1.45 (1.33–1.55)	<0.01
Hemoglobin (g/dL)	11.5 (10.4–12.5)	12.8 (11.8–13.1)	<0.01
Hematocrit (%)	34.5 (32.0–37.9)	38.7 (36.0–40.9)	<0.01
Beta-blocker medication	19 (11.4)	5 (11.4)	0.999
Antiplatelet therapy	29 (17.4)	6 (13.6)	0.654
Anticoagulant therapy	12 (7.2)	4 (9.1)	0.749
Old myocardial infarction	2 (1.2)	1 (2.3)	0.506
Chronic atrial fibrillation	10 (6.0)	1 (2.3)	0.466
Maintenance hemodialysis	0 (0.0)	0 (0.0)	0.999
Pacemaker implantation	1 (0.6)	1 (2.3)	0.371
Moderate to severe AR and/or MR	28 (16.8)	8 (18.2)	0.824
Moderate to severe TR and/or PR	34 (20.4)	6 (13.6)	0.390

AR, aortic regurgitation; MR, mitral regurgitation; TR, tricuspid regurgitation; PR, pulmonic regurgitation.

**Table 2:** Echocardiographic findings of patients with hip fracture and those who underwent total hip arthroplasty (THA).

	Hip fracture (*n* = 167)	THA (*n* = 44)	*P*
End diastolic volume (ml)	68.3 (50.9–83.5)	79.9 (68.5–90.8)	<0.01
End systolic volume (ml)	21.9 (16.6–28.3)	25.2 (21.4–31.9)	<0.01
Ejection fraction (%)	67.0 (64.0–71.0)	67.5 (63.3–72.0)	0.898
Fractional shortening (%)	37.0 (34.0–40.0)	37.0 (34.0–41.0)	0.684
Stroke volume (ml)	45.6 (34.2–55.2)	50.9 (44.2–64.3)	<0.01
Stroke index (ml/m^2^)	33.7 (25.6–40.1)	36.6 (32.0–43.9)	<0.01
Heart rate (beats/min)	78.0 (70.0–86.0)	66.5 (61.3–77.5)	<0.01
Cardiac output (L/min)	3.51 (2.85–4.25)	3.48 (3.12–4.28)	0.273
Cardiac index (L/min/m^2^)	2.60 (2.03–3.11)	2.47 (2.18–2.91)	0.855
E/E′	14.2 (11.7–16.2) (*n* = 120)	11.6 (10.5–16.3) (*n* = 39)	0.092
Inferior vena cava diameter (mm)	12.4 (10.0–14.0) (*n* = 166)	12.5 (10.3–14.0)	0.714

E/E′ indicates the ratio of early mitral valve flow velocity (E) to early diastolic mitral annular velocity (E′), and it correlates with left ventricular filling pressure and diastolic function. A septal E′ was used to determine E/E′ and the numbers of patients whose E/E′ could be measured are presented in each cell. Inferior vena cava diameter of one patient in the hip fracture group could not be measured.

## Data Availability

All relevant data used to support the findings of this study are included within this article. Other datasets are available from the corresponding author upon request.
